# Combination of red ginseng and velvet antler extracts prevents skin damage by enhancing the antioxidant defense system and inhibiting MAPK/AP-1/NF-κB and caspase signaling pathways in UVB-irradiated HaCaT keratinocytes and SKH-1 hairless mice

**DOI:** 10.1016/j.jgr.2024.01.003

**Published:** 2024-01-17

**Authors:** Van-Long Truong, Yeon-Ji Bae, Ji-Hong Bang, Woo-Sik Jeong

**Affiliations:** Food and Bio-industry Research Institute, School of Food Science & Biotechnology, College of Agriculture and Life Sciences, Kyungpook National University, Daegu, Republic of Korea

**Keywords:** Red ginseng extract, Velvet antler extract, Skin protection, UVB radiation

## Abstract

**Background:**

Studies have reported that the combination of two or more therapeutic compounds at certain ratios has more noticeable pharmaceutical properties than single compounds and requires reduced dosage of each agent. Red ginseng and velvet antler have been extensively used in boosting immunity and physical strength and preventing diseases. Thus, this study was conducted to elucidate the skin-protective potentials of red ginseng extract (RGE) and velvet antler extract (VAE) alone or in combination on ultraviolet (UVB)-irradiated human keratinocytes and SKH-1 hairless mice.

**Methods:**

HaCaT cells were preincubated with RGE/VAE alone or in combination for 2 h before UVB (30 mJ/cm^2^) irradiation. SKH-1 mice were orally given RGE/VAE alone or in combination for 15 days before exposure to single dose of UVB (600 mJ/cm^2^). Treated cells and treated skin tissues were collected and subjected to subsequent experiments.

**Results:**

RGE/VAE pretreatment alone or in combination significantly prevented UVB-induced cell death, apoptosis, reactive oxygen species production, and DNA damage in keratinocytes and SKH-1 mouse skins by downregulating mitogen-activated protein kinases/activator protein 1/nuclear factor kappa B and caspase signaling pathways. These extracts also strengthened the antioxidant defense systems and skin barriers in UVB-irradiated HaCaT cells and SKH-1 mouse skins. Furthermore, RGE/VAE co-administration appeared to be more effective in preventing UVB-caused skin injury than these extracts used alone.

**Conclusion:**

Overall, these findings suggest that the consumption of RGE/VAE, especially in combination, offers a protective ability against UVB-caused skin injury by preventing inflammation and apoptosis and enhancing antioxidant capacity.

## Graphical abstract

**Figure:**
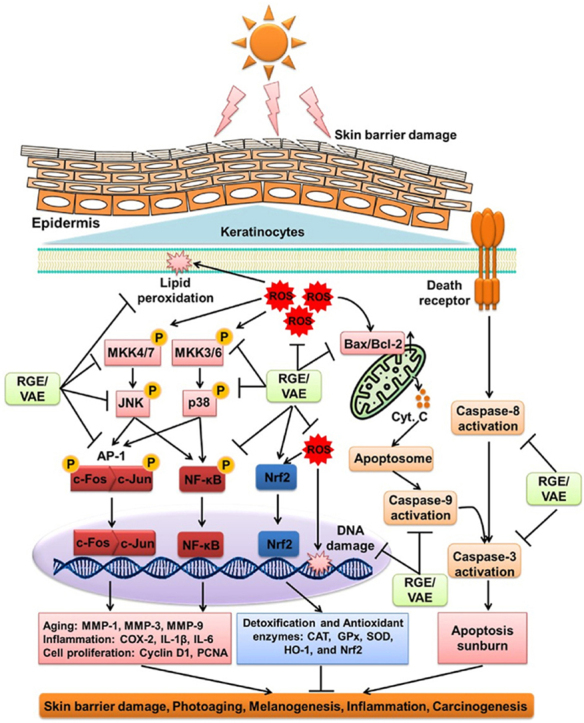

## 1 Introduction

The skin, consisting of the epidermis, dermis, and hypodermis, covers entire external surface of the body, and it is frequently subjected to various environmental insults [1]. Among these, solar ultraviolet (UV) radiation is the most universal damaging environmental factor. The exposure of human to solar radiation, particularly its UVB component, has various adverse effects, including erythema, hyperpigmentation, photoaging, and skin cancer [2].

Numerous natural products are indicated to affect the development of skin structures and their biological functions. Korean red ginseng (*Panax ginseng* Meyer), one of the most popular traditional medicines in Asia, exerts various beneficial effects, such as having anti-fatigue properties, boosting immunity, enhancing memory and cognitive functions of the brain, and regulating blood glucose [[3], [4], [5], [6]]. Recent evidence has indicated the human health benefits of red ginseng consumption, particularly in improving skin conditions [7]. Red ginseng extract (RGE) exerts positive effects on the skin structure [8], and its topical formula can inhibit melanogenesis and prevent skin aging arising from UVB irradiation [9,10]. Red ginseng also suppresses UV-induced cutaneous inflammation and skin cancer both *in vitro* and *in vivo* [11,12].

Similar to Korean red ginseng, velvet antler has been used in folk remedy for body restoration, improving sexual function, and avoiding diseases [[13], [14], [15]]. Recent investigations have demonstrated the positive effects of velvet antler extract (VAE) on the skin structure and dermal microenvironment. VAE stimulates the production of extracellular matrix constituents, such as type 1 collagen, fibronectin, and elastin in dermal fibroblasts, contributing to the conservation of skin structure [16]. A clinical trial showed VAE-containing sponge microspicule cream decreased the melanin and erythema levels and simultaneously increased elasticity and moisturization in human skin, thereby improving skin properties without skin irritation [17]. In addition, applying VAE-containing serum on the human scalp remarkably stimulated hair growth and increased hair pigment and skin hydration without skin irritation [18]. While RGE has been extensively studied and proven efficacy in mitigating UV-induced skin damage, very few studies has addressed on the impact of VAE on skin health, and the mechanisms underlying its potential benefits remain unknown. Thus, further studies are required to contribute to clarifying the benefits of VAE on skin health and comparing its effects with those of RGE.

Recent studies have reported that the combination of two or more therapeutic agents at certain ratios has more pronounced pharmacological properties than single compounds used alone and requires reduced dosage of each agent to avoid toxicity and undesired adverse effects [19]. Korean red ginseng is often mixed with other compounds to produce combined or synergistic effects on certain pathological conditions. A combination of RGE and *Epimedium koreanum* Nakai synergistically inhibited lipopolysaccharide-triggered inflammation in RAW 264.7 macrophages and synergistically mitigated ulcerative colitis in mice [20]. The mixture (KTNG0345) of RGE, *Torilis fructus*, and *Corni fructus* extracts exhibited anti-wrinkle ability in UVB-exposed SKH-1 mice [21]. Furthermore, a mixture of RGE and VAE was reported to be safe and exhibited no adverse effects on rats after repeated oral administrations of 2000 mg/kg/day for 13 weeks [22]. Inspired by these findings, we hypothesized that a combination of RGE with VAE could yield beneficial effects on skin health.

Despite accumulating evidence that the consumption of Korean red ginseng or velvet antler can improve skin health, the protective effects of RGE and/or VAE, especially in combination, against UVB-induced skin injury remain unknown. Therefore, this study was conducted to elucidate the protective potentials of RGE/VAE alone or in combination against skin damage in UVB-irradiated human keratinocytes and SKH-1 hairless mice.

## 2 Materials and methods

### 2.1 Materials

RGE and VAE used in this study were supplied by the Korea Ginseng Corporation (Daejeon, Republic of Korea). The bioactive compositions of RGE are the ginsenosides Rg1, Re, Rb1, Rc, Rb2, and Rd [4]. The main constituents present in VAE include amino acids (e.g., glycine, alanine, and serine), minerals (e.g., Zn, Mn, and Ca), proteins and peptides (e.g., collagen, crude proteins, and growth factors), saccharides (e.g., sialic acid, uronic acid, and glycosaminoglycans), and lipids and polyamines (e.g., unsaturated fatty acids, prostaglandins, progesterone, and testosterone) [13,23]. Eosin, formalin, 2′,7′-dichlorofluorescein diacetate (DCHF-DA), vitamin C, and hematoxylin were purchased from Sigma-Aldrich (St. Louis, MO, USA). The primary and secondary antibodies were acquired from Santa Cruz Biotechnology (Santa Cruz, CA, USA), Cell Signaling Technology (Boston, MA, USA), Abcam (Cambridge, UK), R&D Systems (Minneapolis, MN, USA), and Thermo Scientific (Waltham, MA, USA).

### 2.2 Cell culture and UVB irradiation

Spontaneously immortalized human keratinocytes HaCaT cells, obtained from Cell Line Service (Heidelberg, Germany), were cultured in Dulbecco's modified Eagle's medium (Cytiva, Marlborough, MA, USA) containing 10 % fetal bovine serum and 1 % penicillin/streptomycin solution at 37 °C in humidified incubator with 5 % CO_2_. Before treatment, the cells were starved in a serum-free medium for 24 h. The cells were washed twice with phosphate-buffered saline and then irradiated with UVB at a dose of 30 mJ/cm^2^ [2]. UVB irradiation was conducted using a Bio-Link 312 Crosslinker (Vilber Lourmat, Torcy, France), emitting peak wavelength at 312 nm.

### 2.3 Cell viability

HaCaT cells were pretreated with various RGE/VAE concentrations alone or in combination for 2 h before UVB (30 mJ/cm^2^) irradiation. Then, the cells were incubated with samples for an additional 24 h. Cell viability was determined using a Cell Counting Kit assay kit (Sigma).

### 2.4 Dtermination of reactive oxygem species (ROS) accumulation

The ROS formation was estimated using a permeable probe, DCHF-DA. Briefly, HaCaT cells were pretreated with RGE/VAE alone or in combination for 2 h before UVB (30 mJ/cm^2^) irradiation. Then, the cells were incubated with 20 μM DCFH-DA for 30 min in a 37 °C humidified incubator. After washing out excessive probes, fluorescence (excitation/emission wavelength of 485/528 nm) was read immediately using a microplate reader.

### 2.5 Animal experiment

Female SKH-1 hairless mice (six-week-old, 20–22 g) were acquired from Hana Biotech (Gyeonggi-do, Republic of Korea). Animals were housed under standard conditions of 12-h light/dark cycle, 22 °C ± 2 °C, and 50 % ± 5 % humidity. The animals were given a laboratory standard diet and water *ad libitum*. All experimental procedures were approved by the Institutional Animal Care and Use Committee of Kyungpook National University (No. KNU 2021-182). Animals were randomly divided (n = 6 each) into the control group, UVB group, UVB + RGE (100 mg/kg) group, UVB + VAE (100 mg/kg) group, UVB + RGE (50 mg/kg) + VAE (50 mg/kg) group, and UVB + vitamin C (200 mg/kg) group. The mice were orally given RGE/VAE alone or in combination for 15 days before exposure to a single dose of UVB (600 mJ/cm^2^). The control group was given distilled water as a vehicle, whereas the positive control group was orally administered with vitamin C (200 mg/kg) for 15 days before UVB irradiation. After 1 h from the last dose, the animals, except for the control group, were subjected to single UVB (600 mJ/cm^2^) irradiation using a Bio-Link Crosslinker (Vilber Lourmat). The doses of RGE, VAE, and vitamin C were chosen according to previous studies [[24], [25], [26]], while the intensity of UVB radiation was based on a previous study [27]. After 12 h of UVB exposure, the mice were euthanized, and the back skin tissues were collected and kept in −80 °C for subsequent experiments.

### 2.6 Histopathological evaluation

The skin biopsies were kept in 10 % neutral formalin and fixed in paraffin. Sections (5 μm) were then deparaffinized and stained with hematoxylin and eosin (H&E). The H&E-stained sections were scanned under a light microscope, and representative images were taken by a digital microscope (Paxcam, Iowa, IL, USA).

### 2.7 Immunohistochemical analysis

For immunostaining, the deparaffinized sections were introduced to antigen retrieval by heating in 10 mM citrate buffer (pH 6.0) in a microwave for 15 min. After blocking, the sections were hybridized with specific primary antibodies at 4 °C overnight and then incubated with appropriate horseradish peroxidase-conjugated secondary antibodies at 4 °C for 3 h. The peroxidase-binding sites were detected by incubating with 3,3′-diaminobenzidine tetrahydrochloride. Eventually, tissue sections were counterstained with Mayer's hematoxylin (Sigma), and immunostained images were taken using a digital microscope (Paxcam).

### 2.8 Terminal deoxynucleotidyl transferase dUTP nick end-labeling (TUNEL) assay

Apoptotic cells in mouse skin tissues were detected using the TUNEL assay kit (Roche, Switzerland). Representative images were taken using a digital microscope (Paxcam).

### 2.9 Antioxidant enzyme activity and lipid peroxidation assays

Treated cells and skin tissues were homogenized in suitable lysis buffers, and lysates were then centrifuged at 13,000 rpm, 4 °C for 15 min to collect supernatants. Activities of antioxidant enzymes, namely, catalase (CAT), glutathione peroxidase (GPx), and superoxide dismutase (SOD), were measured using colorimetric assay kits (Biovision, Waltham, MA, USA). Lipid peroxidation in skin tissues was evaluated by measuring the amount of malondialdehyde using a colorimetric assay kit (Biovision).

### 2.10 Western blot analysis

Proteins from treated cells and skin biopsies were extracted with RIPA buffer (Cell Signaling) and total protein concentration was quantified using a Pierce™ BCA Protein Assay Kit (Thermo Fisher Scientific). The protein samples were fractionated on sodium dodecyl-sulfate polyacrylamide gels and followed by electro-blotting onto Immobilon®-P polyvinylidene fluoride membranes (Millipore, Burlington, MA, USA). After blocking with 5 % skim milk for 2 h at room temperature, the membranes were probed with specific primary antibodies at 4 °C overnight and subsequently hybridized with appropriate secondary antibodies at 4 °C for 3 h. Finally, protein bands were visualized with EzWestLumi Plus Reagent (Atto, Tokyo, Japan).

### 2.11 Statistical analysis

Data were depicted as the mean ± standard deviation (SD) and statistical analysis was operated using GraphPad Prism version 8.0 (GraphPad, San Diego, CA, USA). A statistically significant difference was assessed by the one-way or two-way analysis of variance (ANOVA) followed by Tukey's post-hoc test. Values of * *p* < 0.05, **p < 0.01, and ***p < 0.001 were considered statistically significant.

## 3 Results and discussion

### 3.1 RGE/VAE and their combination prevent UVB-induced apoptotic cell death in HaCaT cells and SKH-1 mouse skins

**Fig. 1:**
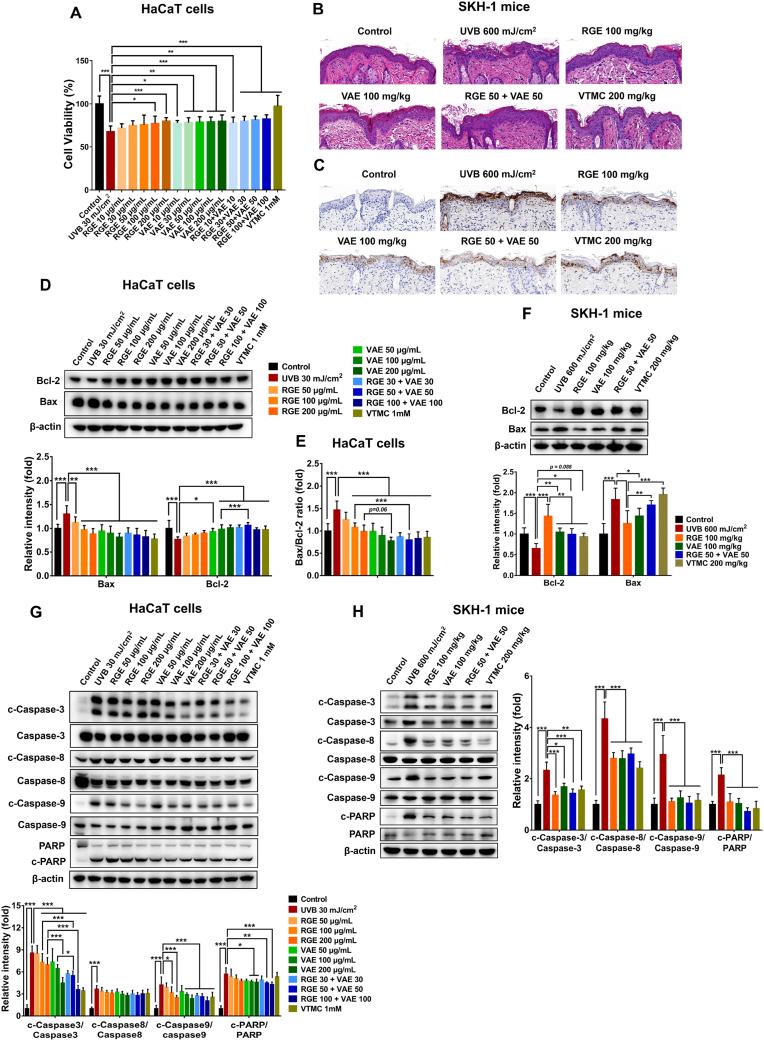
Protective abilities of RGE/VAE and their combination against UVB-induced apoptotic cell death in HaCaT cells and SKH-1 hairless mice. (**A**) HaCaT cell viability. (**B**) H&E staining of mouse skins. (**C**) TUNEL staining of mouse skins. (**D**) Expressions of Bcl-2 and Bax in HaCaT cells. (**E**) Bcl-2/Bax ratio in HaCaT cells. (**F**) Expressions of Bcl-2 and Bax in mouse skin tissues. (**G** and **H**) Expressions of caspase-3, cleaved caspase-3, caspase-8, cleaved caspase-8, caspase-9, cleaved caspase-9, PARP, and cleaved PARP in HaCaT cells and mouse skins, respectively. Data are presented as the mean ± SD. Statistically significant differences were determined by one-way or two-way ANOVA followed by Tukey post-hoc test (*p < 0.05, **p < 0.01, and ***p < 0.001).

To elucidate the cytoprotective abilities of RGE/VAE alone or in combination against UVB-caused HaCaT cell death, the relative percentages of viable keratinocytes, the dominant cell type present in the epidermis, were determined. As shown in Fig. 1A, UVB (30 mJ/cm^2^) exposure to HaCaT cells reduced cell viability by approximately 40 %, compared with non-irradiated cells. However, RGE/VAE pretreatment significantly prevented UVB-induced cell death. No significant difference in the protective abilities of RGE and VAE was observed. In addition, the low-dose RGE/VAE combination protected human keratinocytes against UVB-induced cytotoxicity, which was equivalent to or more pronounced than individual extracts at full doses.

To investigate the skin-protective abilities of RGE/VAE alone or in combination, UVB-induced SKH-1 hairless mice were used as a skin model. Histomorphological analysis showed that UVB exposure markedly augmented apoptotic sunburned cells in mouse skins, compared with the control, whereas the RGE/VAE-treated mice exhibited a considerable reduction in UVB-induced sunburned cells (Fig. 1B). In addition, the co-administration of RGE and VAE decreased apoptotic cells in UVB-exposed mouse skins. Apoptosis is an orchestrated cellular process indicated by morphological alterations, such as cell shrinkage, pyknosis, karyorrhexis, and formation of apoptotic bodies [2,28]. UVB is a major environmental agent causing apoptotic cell death in skin cells. In this study, TUNEL staining results indicated that UVB-irradiated mouse skins showed higher number of apoptotic cells in the epidermis compared with the control group (Fig. 1C). RGE/VAE supplementation prevented UVB-induced development of apoptotic cells. No difference in the anti-apoptotic activities of RGE and VAE was noted, whereas the RGE/VAE combination appeared to be more potent than these extracts used alone.

UVB-induced apoptosis is a complicated process associated with multiple pathways, such as DNA damage (intrinsic pathway), death receptor activation (extrinsic pathway), and ROS formation independently and/or synergistically [29,30]. As members of the large Bcl-2 family, anti-apoptotic Bcl-2 and pro-apoptotic Bax proteins regulate mitochondria-related apoptotic process [31]. Bcl-2 suppresses apoptosis by preventing Bax activity, whereas Bax promotes cell death by increasing the permeability of mitochondrial membrane, leading to the release of apoptogenic factors and subsequently the activation of apoptotic cascades [32]. The Bax/Bcl-2 ratio is considered as a prognostic marker determining the susceptibility of cell to apoptosis. In this study, UVB-exposed HaCaT cells exhibited a significant upregulation of Bax expression in a concomitant reduction of Bcl-2 level, resulting in a shift in the Bax/Bcl-2 ratio that favors apoptosis. However, RGE/VAE pretreatment alone or in combination significantly reversed these UVB-induced events (Fig. 1D and E). Furthermore, low-dose RGE/VAE combination also exerted inhibitory effects on UVB-mediated apoptosis, which were equivalent to those of RGE and VAE alone at full doses. Similarly, aberrant expressions of Bcl-2 and Bax in UVB-exposed mouse skins were normalized by RGE/VAE supplementation (Fig. 1F).

Regarding the extrinsic pathway, caspase cascades, including initiators (caspase-8, caspase-9, and caspase-10) and executors (caspase-3, caspase-6, and caspase-7), importantly contribute to apoptotic signal transduction [33]. Caspase-3 is a central effector caspase that is involved in both intrinsic and extrinsic pathways and plays a crucial role in UV-mediated apoptosis [34,35]. This study confirmed that UVB irradiation markedly promoted a caspase-dependent apoptotic pathway by increasing the levels of cleaved forms of caspase-3, caspase-8, caspase-9, and poly-ADP ribose polymerase (PARP). By contrast, RGE/VAE pretreatment significantly inhibited UVB-induced active forms of these caspases in both human keratinocytes and mouse skins (Fig. 1G and H). It is noteworthy that compared to the RGE, VAE exhibited a greater efficacy in reducing cleaved-caspase-3 than RGE, and a combination of RGE with VAE produced a synergistically inhibitory effect on caspase-3 activation in UVB-exposed HaCaT cells. However, these effects were not observed in UVB-irradiated hairless mice. Overall, these observations suggest that RGE/VAE alone or in combination prevents UVB-caused apoptotic cell death in skin cells, possibly through regulating Bcl-2 family members and interfering caspase cascade activation.

### 3.2 RGE/VAE and their combination inhibit UVB-induced inflammatory response in HaCaT cells and SKH-1 mouse skins

Although keratinocytes are responsible for producing structural components and maintaining barrier function of the epidermis, these cells are believed to participate in inflammatory and immunomodulatory actions [36]. In response to skin injury, UV radiation, and chemicals, keratinocytes produce various pro-inflammatory cytokines and mediators, provoking inflammatory skin diseases. Cyclooxygenase-2 (COX-2), responsible for the generation of prostaglandins, plays a critical role in UVB-induced acute inflammation and is responsible for clinical erythema. UVB-induced COX-2 expression also stimulates the production of cytokines such as interleukin (IL)-6 and IL-8 during the progression phase of the inflammatory process [36,37]. COX-2 suppression mitigates UV-induced cutaneous inflammation and skin carcinogenesis [38,39]. In addition, UVB-induced cytokines and chemokines, such as IL-1β, tumor necrosis factor (TNF)-α, and monocyte chemoattractant protein-1 from keratinocytes trigger inflammatory response and induce inflammatory cell infiltration to injured skin tissues [37]. Therefore, the management of inflammation is imperative for the prevention of UVB-mediated skin disorders.

**Fig. 2:**
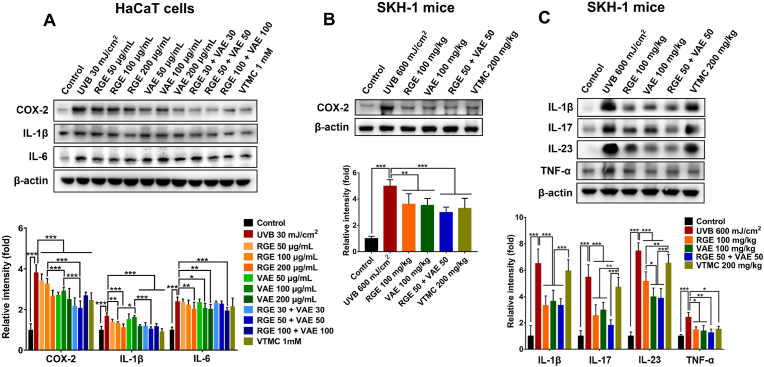
Anti-inflammatory effects of RGE/VAE and their combination in UVB-irradiated HaCaT cells and SKH-1 hairless mice. (**A**) Expressions of COX-2, IL-1β, and IL-6 in HaCaT cells. (**B**) COX-2 expression in mouse skins. (**C**) Expressions of IL-1β, IL-17, IL-23, and TNF-α in mouse skins. Data are presented as the mean ± SD. Statistically significant differences were determined by one-way or two-way ANOVA followed by Tukey post-hoc test (*p < 0.05, **p < 0.01, and ***p < 0.001).

In this study, the anti-inflammatory potentials of RGE/VAE alone or in combination were examined using UVB-exposed HaCaT cells and SKH-1 mice. UVB irradiation dramatically increased the levels of COX-2 in keratinocytes, whereas RGE/VAE pretreatment significantly reduced the expression of this enzyme (Fig. 2A). RGE/VAE also abolished the UVB-induced production of IL-1β and IL-6 in keratinocytes (Fig. 2A). In UVB-irradiated keratinocytes, VAE demonstrated greater inhibition of COX-2 expression compared to RGE, whereas RGE exhibited pronounced efficacy in attenuating IL-1β expression compared to VAE at the same concentration. Notably, the low-dose RGE/VAE combination also considerably downregulated the levels of inflammatory factors, which was equivalent or more pronounced than that produced by RGE or VAE alone at full doses, suggesting a synergistically anti-inflammatory activity of RGE/VAE combination.

The anti-inflammatory effects of RGE/VAE alone or in combination were confirmed in UVB-irradiated SKH-1 hairless mice. As indicated in Fig. 2B, orally administered RGE/VAE significantly reduced COX-2 expression in UVB-exposed mouse skin tissues, and no statistically significant difference was observed between the RGE and VAE groups. UVB-induced generation of IL-β, IL-17, IL-23, and TNF-α in mouse skin tissues was considerably prevented by RGE/VAE supplementation (Fig. 2C). Furthermore, the anti-inflammatory abilities of RGE/VAE combination were similar or superior to those of RGE/VAE alone or vitamin C. Overall, these observations suggest that RGE/VAE supplementation, especially in combination, effectively mitigates UVB-induced skin inflammation.

### 3.3 RGE/VAE and their combination regulate AP-1, NF-κB, and MAPK signaling pathways in UVB-irradiated HaCaT cells and SKH-1 mouse skins

**Fig. 3:**
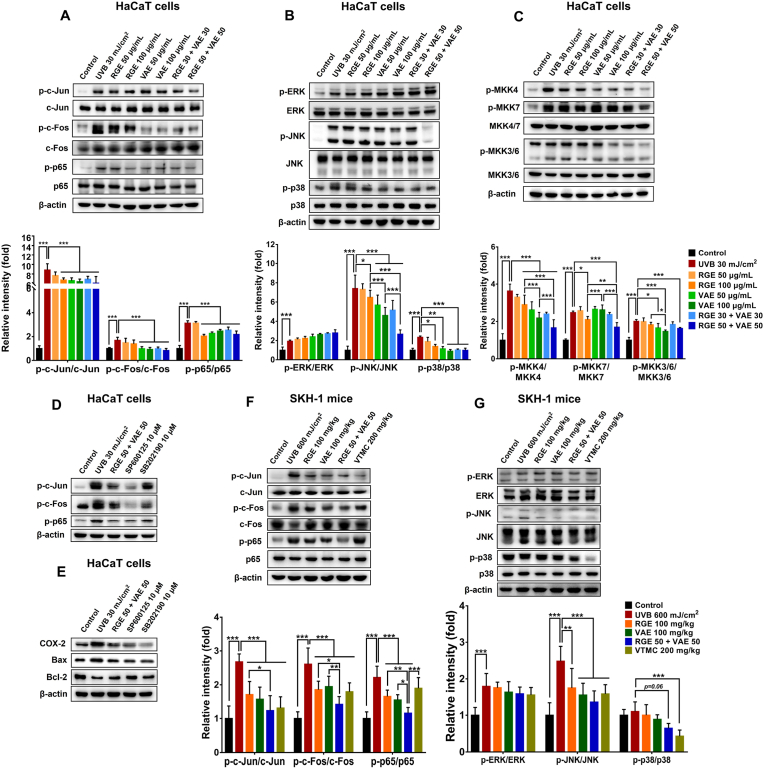
Effects of RGE/VAE and their combination on AP-1, NF-κB, and MAPK signaling pathways in UVB-irradiated HaCaT cells and SKH-1 hairless mice. (**A**) Phosphorylation levels of c-Jun, c-Fos, and p65 in HaCaT cells. (**B**) Phosphorylation levels of ERK, JNK, and p38 MAPK in HaCaT cells. (**C**) Phosphorylation levels of MKK4/7 and MKK3/6 in HaCaT cells. (**D** and **E**) The cells were pretreated with RGE/VAE or specific inhibitors (SB202190 for p38 MAPK and SP600125 for JNK) for 2 h before UVB radiation, and the cells were continuously incubated with inhibitors for 30 min or 12 h. Phosphorylation levels of (**D**) c-Jun, c-Fos, and p65 and (**E**) COX-2, Bax, and Bcl-2 in HaCaT cells. (**F**) Phosphorylation levels of c-Jun, c-Fos, and p65 in mouse skins. (**G**) Phosphorylation levels of ERK, JNK, and p38 MAPK in mouse skins. Data are presented as the mean ± SD. Statistically significant differences were determined by one-way or two-way ANOVA followed by Tukey post-hoc test (*p < 0.05, **p < 0.01, and ***p < 0.001).

UVB radiation triggers responses in epidermal keratinocytes, including the production of inflammatory cytokines and apoptotic mediators by activating various signaling pathways. Various transcription factors are hyperactivated in response to UVB radiation, including activator protein-1 (AP-1) and nuclear factor kappa B (NF-κB), which regulate the expressions of many genes associated with cell proliferation, apoptosis, and inflammation [40,41]. To better understand the possible skin-protective mechanisms of RGE/VAE, the effects of RGE/VAE alone or in combination on several signaling pathways in UVB-exposed HaCaT cells and SKH-1 mice were examined. Accordingly, UVB exposure activated AP-1 and NF-κB pathways in the keratinocytes by enhancing the phosphorylation of c-Jun, c-Fos, and p65 (Fig. 3A). However, RGE/VAE pretreatment significantly inhibited UVB-induced AP-1 and NF-κB activation, as evidenced by reduced phosphorylation of these proteins (Fig. 3A). There was no significant difference between RGE and VAE groups, while RGE/VAE combination was more potent than these extracts used alone.

MAPKs, composed of extracellular-related kinase (ERK), c-Jun N-terminal kinase (JNK), and p38 MAPK, participate in transmitting extracellular stimuli to intracellular signals, which promote cellular events such as proliferation, inflammation, and apoptosis [42]. Recent studies have demonstrated that UVB activates all three MAPKs and several upstream kinases, including MAPK kinases (MAPKKs); among them, JNK and p38 MAPK are implicated in UVB-induced inflammation and mitochondria-mediated apoptosis [1,42,43]. Moreover, these kinases are also upstream activators of AP-1 and NF-κB; their UVB-enhanced activation leads to hyperactivation of these transcription factors and consequently more robust induction of genes related to skin inflammation and photoaging [36,44].

In this study, UVB promoted MAPK signals by elevating the levels of p-ERK, p-JNK, and p-p38 MAPK in HaCaT keratinocytes (Fig. 3B), whereas the pretreatment of keratinocytes with RGE/VAE alone or in combination significantly suppressed UVB-induced phosphorylation of JNK and p38 MAPK, but not ERK (Fig. 3B). We also elucidated the effects of RGE/VAE on MAPKKs, the upstream kinase of MAPKs, as RGE/VAE affects MAPK phosphorylation. The results showed that RGE/VAE alone and in combination significantly reduced levels of p-MKK3/6 and p-MKK4/7 in UVB-irradiated keratinocytes (Fig. 3C). Notably, compared to RGE, VAE exhibited a greater effectiveness in blocking the JNK signaling pathway in UVB-irradiated HaCaT cells. Furthermore, the combination of RGE with VAE produced a superior inhibitory effect of this pathway than individual extracts, implying a synergistic effect.

To further investigate the involvement of JNK and p38 MAPK signals in the anti-apoptotic and anti-inflammatory mechanisms of RGE and VAE, HaCaT cells were preincubated with RGE/VAE or specific inhibitors (SB202190 for p38 MAPK and SP600125 for JNK) before UVB irradiation. The results showed that JNK (SP600125) and p38 MAPK (SB202190) inhibitors remarkably inhibited UVB-stimulated AP-1 and NF-κB activation by attenuating c-Jun, c-Fos, and p65 phosphorylation in keratinocytes, which were equivalent to the effects of RGE/VAE (Fig. 3D). In addition, the suppression of JNK and p38 MAPK pathways remarkably attenuated the COX-2 and Bax levels but increased Bcl-2 expression (Fig. 3E). The p38 MAPK inhibitor effectively prevented the UVB-induced inflammatory response, whereas the JNK inhibitor effectively prevented the UVB-induced apoptosis process.

The effects of RGE/VAE on AP-1, NF-κB, and MAPK signaling pathways were further confirmed in UVB-exposed SKH-1 mice. In the line with the *in vitro* results, UVB-induced AP-1 and NF-κB activation was suppressed in RGE/VAE-pretreated mice by reducing the levels of p-c-Jun, p-c-Fos, and p-p65 (Fig. 3F). Despite the comparable inhibitory effects of RGE and VAE, the effects of RGE/VAE combination were more pronounced than those when the compounds were used alone. Furthermore, RGE/VAE also reduced the UVB-caused phosphorylation of JNK and p38 MAPK in the mouse skin (Fig. 3G). Overall, these findings suggest that RGE/VAE, especially in combination, may attenuate the UVB-induced skin injury by suppressing the AP-1, NF-κB, JNK, and p38 MAPK signaling pathways.

### 3.4 RGE/VAE and their combination inhibit UVB-induced oxidative stress in HaCaT cells and SKH-1 mouse skin

The skin is equipped with various antioxidant enzymes and small antioxidant molecules that can protect against ROS. However, UVB radiation provokes excessive ROS production that defeats the antioxidant capacity of the skin, resulting in oxidative stress [1,2]. UVB-induced ROS overproduction can damage cellular biomolecules, such as DNA, proteins, and lipids. Ultimately, accumulating oxidative damage is associated with various cutaneous pathological conditions such as sunburn, photoaging, and photocarcinogenesis [43,45,46]. Therefore, the supplementation of natural antioxidants has become a primary prevention strategy to attenuate the detrimental effects of ROS on the skin.

**Fig. 4:**
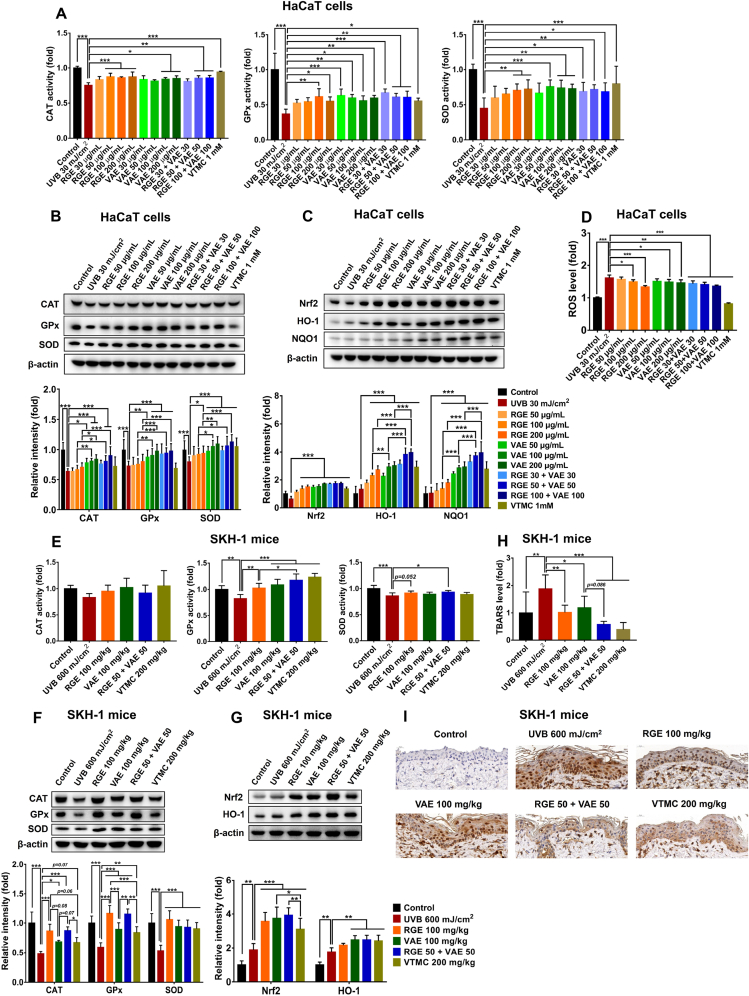
Antioxidant effects of RGE/VAE and their combination in UVB-irradiated HaCaT cells and SKH-1 hairless mice. (**A** and **B**) Activities and protein expressions of CAT, GPx, and SOD in HaCaT cells. (**C**) Protein expressions of Nrf2, HO-1, and NQO-1 in HaCaT cells. (**D**) ROS production in HaCaT cells. (**E** and **F**) Activities and protein expressions of CAT, GPx, and SOD in mouse skins. (**G**) Protein expressions of Nrf2 and HO-1 in mouse skins. (**H**) Lipid peroxidation (TBARS level) in mouse skins. (**I**) 8-OHdG immunostaining in mouse skins. Data are presented as the mean ± SD. Statistically significant differences were determined by one-way or two-way ANOVA followed by Tukey post-hoc test (*p < 0.05, **p < 0.01, and ***p < 0.001).

To explore whether the preventive effects of RGE/VAE could be relevant to their antioxidant abilities, the inductive effects of RGE/VAE alone or in combination on cytoprotective genes in UVB-treated HaCaT cells were examined. The exposure of keratinocytes to UVB significantly decreased the activities of primary antioxidant enzymes, including CAT, GPx, and SOD, compared to the control (Fig. 4A). However, RGE/VAE pretreatment restored the activities of these enzymes. RGE/VAE also considerably enhanced the levels of CAT, GPx, and SOD in UVB-irradiated HaCaT cells, and the efficacy of VAE appeared to be more potent than that of RGE (Fig. 4B). Furthermore, the low-dose RGE/VAE combination produced similar or superior antioxidant activities than individual extracts at full doses.

Besides primary antioxidant enzymes, nuclear factor erythroid 2–related factor 2 (Nrf2)-mediated phase II enzymes contribute to the protection from solar UV radiation-induced cutaneous photodamage and cell dysfunction. Nrf2 is a master regulator of phase II detoxification and antioxidant enzymes, such as heme oxygenase-1 (HO-1), NAD(P)H quinone oxidoreductase 1 (NQO-1), and glutathione reductase, which counteract the UV-induced oxidative damage [47,48]. By contrast, the detrimental effects of UV radiation are accelerated in Nrf2 knockout mice [49,50]. Recent studies have also shown that the activation of the Nrf2 pathway is required for the cytoprotective, anti-apoptotic, anti-inflammatory, and antioxidant activities of various botanical compounds in UVB-irradiated keratinocytes and mice [51,52]. In this study, both RGE and VAE upregulated the expressions of Nrf2, HO-1, and NQO-1 in UVB-irradiated keratinocytes, and the efficacy of VAE appeared to be better than that of RGE (Fig. 4C). Notably, the low-dose RGE/VAE combination produced equivalent or superior inductive effects than the individual extracts at full doses. Consequently, UVB-induced ROS formation in keratinocytes was markedly decreased by RGE/VAE pretreatment alone or in combination (Fig. 4D).

The antioxidant capacities of RGE/VAE alone or in combination were further confirmed in UVB-exposed SKH-1 mice. Consistent with the *in vitro* results, RGE/VAE supplementation improved antioxidant defense systems in the mouse skin by restoring the activities and expressions of CAT, GPx, and SOD (Fig. 4E and F). In addition, RGE/VAE administration also enhanced the expressions of Nrf2 and HO-1 in UVB-exposed mouse skins (Fig. 4G). UVB irradiation to the skin results in ROS formation that attacks cellular components such as DNA, lipids, and proteins. Lipid peroxidation, an oxidative stress-caused event, has a highly deleterious effect on cell membrane structure and function, ultimately resulting in cell death [53,54]. UVB not only directly causes DNA modifications but also indirectly provokes oxidative damage in DNA, leading to the generation of 8-OHdG adduct, which critically contributes to UVB-mediated photocarcinogenesis [2]. Recent studies have indicated that botanical agents can reduce UVB-induced 8-OHdG generation and lipid peroxidation in keratinocytes and mouse epidermis, preventing the harmful effects of UV radiation [2,35,43,52]. In this study, UVB-induced lipid peroxidation was markedly reduced in the RGE/VAE groups, as illustrated by the TBARS level (Fig. 4H). Immunostaining analysis results also showed significant decreases in the levels of stained 8-OHdG, a biomarker of UVB-induced oxidative damage to DNA, in RGE/VAE groups (Fig. 4I). Interestingly, the antioxidant abilities of RGE/VAE combination in UVB-irradiated SKH-1 mice appeared to be more pronounced than those of the extracts used alone.

Overall, these results suggest that RGE/VAE supplementation, especially in combination, may prevent UVB-induced skin damage by enhancing antioxidant defense systems.

### 3.5 RGE/VAE and their combination enhance skin-barrier proteins in UVB-irradiated HaCaT cells and SKH-1 mouse skins

The skin-barrier serves as a vital physical barrier against external factors, such as pathogens, particle matter, and UV radiation and maintains skin homeostasis [55]. UVB radiation can reduce skin-barrier function by altering the cornified envelope, which maintains the structural and mechanical integrity of the stratum corneum. The function of the cornified envelope depends on the interconnections among transglutaminase-crosslinked proteins, including filaggrin, loricrin, and involucrin. UVB irradiation can cause the desquamation of keratinocytes, leading to the reduced synthesis of skin-barrier proteins. Consequently, impaired skin-barrier function may increase the risk of pathogen intake and subsequently cause cutaneous inflammation. In turn, inflammation, characterized by the overproduction of pro-inflammatory cytokines and mediators that cause tissue destruction, provokes skin-barrier dysfunction [55,56]. Therefore, maintaining the expressions of skin-barrier proteins importantly contributes to enhancing skin health and protecting the skin from harmful stimuli in the environment.

**Fig. 5:**
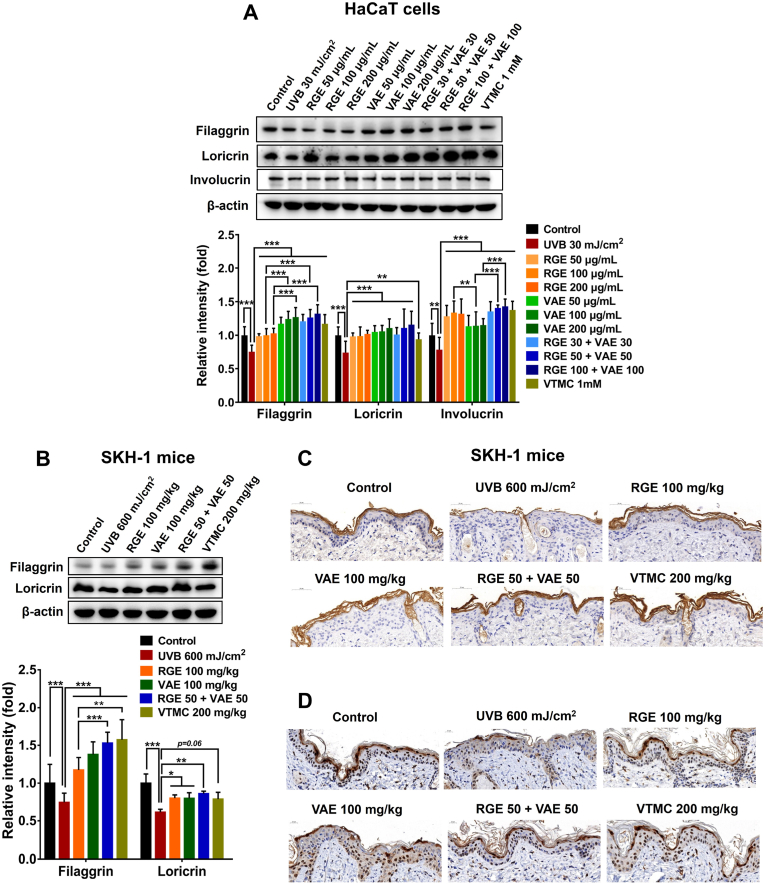
Effects of RGE/VAE and their combination on the level of skin-barrier proteins in UVB-irradiated HaCaT cells and SKH-1 hairless mice. (**A**) Protein expressions of filaggrin, loricrin, and involucrin in HaCaT cells. (**B**) Protein expressions of filaggrin and loricrin in mouse skins. (**C** and **D**) Filaggrin and loricrin immunostaining in mouse skins, respectively. Data are presented as the mean ± SD. Statistically significant differences were determined by one-way or two-way ANOVA followed by Tukey post-hoc test (*p < 0.05, **p < 0.01, and ***p < 0.001).

In this study, UVB irradiation reduced the levels of filaggrin, loricrin, and involucrin in human keratinocytes, suggesting an increased risk of apparent skin-barrier damage (Fig. 5A). However, RGE/VAE pretreatment considerably restored the levels of these proteins, and RGE appeared to be better than VAE (Fig. 5A). Furthermore, RGE/VAE combination produced similar or superior effects than individual compounds, implying a synergistic effect.

In line with the *in vitro* results, RGE/VAE administration prevented UVB-induced loss of filaggrin and loricrin, and their combination appeared to be more potent (Fig. 5B). Immunostaining analysis confirmed a significant recovery of filaggrin and loricrin in the mouse skins of the RGE/VAE groups, compared with the UVB-irradiated group (Fig. 5C and D). Taken together, these observations propose that RGE/VAE consumption, particularly in combination, may improve skin-barrier and thereby prevents UVB-caused skin injury.

In conclusion, this study demonstrates that RGE/VAE supplementation protects skin against the detrimental effects of UVB radiation. RGE/VAE alone or in combination inhibits UVB-caused cell death and apoptosis by preventing ROS accumulation and reducing cleaved forms of caspase-3, caspase-8, caspase-9, and PARP, as well was Bax/Bcl-2 ratio. These compounds also inhibited UVB-induced inflammatory response in human keratinocytes and SKH-1 hairless mice by regulating AP-1, NF-κB, JNK, and p38 MAPK signaling pathways. In addition, the skin-protective effects of RGE/VAE are associated with their strong antioxidant activity, as evidenced by enhanced levels of CAT, GPx, SOD, Nrf2, and HO-1, as well as reduced levels of TBARS and 8-OHdG. Furthermore, RGE/VAE prevents the harmful effects of UVB radiation by enhancing skin-barrier proteins, which in turn may protect the cellular components against UVB-induced damage. These findings provide a basis for the photochemopreventive properties of RGE and VAE and propose that RGE/VAE consumption, particularly in combination, may be useful agents for the development of functional food related to human skin health.

## Institutional review board statement

The animal study protocol was approved by the Institutional Animal Care and Use Committee of Kyungpook National University (No. KNU 2021-182, Date 2021/10/15).

## CRediT authorship contribution statement

**Van-Long Truong:** Conceptualization, Methodology, Software, Validation, Formal analysis, Investigation, Resources, Data curation, Writing – original draft, preparation, Writing – review & editing. **Yeon-Ji Bae:** Methodology, Software, Formal analysis, Investigation, Visualization. **Ji-Hong Bang:** Supervision, Project administration, Funding acquisition, and. **Woo-Sik Jeong:** Supervision, Project administration, Funding acquisition.

## Declaration of competing interest

The authors declare no conflict of interest.
